# Age and *Giardia intestinalis* Infection Impact Canine Gut Microbiota

**DOI:** 10.3390/microorganisms9091862

**Published:** 2021-09-02

**Authors:** Anne-Sophie Boucard, Myriam Thomas, Wilfried Lebon, Bruno Polack, Isabelle Florent, Philippe Langella, Luis G. Bermúdez-Humarán

**Affiliations:** 1 Université Paris-Saclay, INRAE, AgroParisTech, Micalis Institute, 78350 Jouy-en-Josas, France; Anne-Sophie.Boucard@inrae.fr (A.-S.B.); philippe.langella@inrae.fr (P.L.); 2 Anses, INRAE, Ecole Nationale Vétérinaire d’Alfort, UMR BIPAR, Laboratoire de Santé Animale, 94700 Maisons-Alfort, France; myriam.thomas@anses.fr (M.T.); bruno.polack@vet-alfort.fr (B.P.); 3 Boehringer Ingelheim Animal Health France, 29 Avenue Tony Garnier, 69007 Lyon, France; Wilfried.LEBON@boehringer-ingelheim.com; 4 UMR 7245, Muséum National d’Histoire Naturelle, Centre National de la Recherche Scientifique, Sorbonne Universités, 75005 Paris, France; isabelle.florent@mnhn.fr

**Keywords:** *Giardia intestinalis*, giardiosis, dogs, gut microbiota, age, intestinal inflammation

## Abstract

*Giardia intestinalis* is a flagellated protozoan responsible for giardiosis (also called giardiasis in humans), the most prevalent and widespread parasitic infection in humans and mammals worldwide. The intestinal microbiota is highly diverse and any alteration in its composition may impact on the health of the host. While studies on the mouse model of giardiosis described the role of the gut microbiota in host susceptibility to infection by the parasite, little is known about the gut microbiota during natural infections in dogs and particularly in puppies. In this study, we monitored naturally *G. intestinalis*-infected puppies for 3 months and quantified cyst excretion every 2 weeks. All puppies remained subclinically infected during the sampling period as confirmed by fecal examination. In parallel, we performed 16S Illumina sequencing of fecal samples from the different time points to assess the impact of *G. intestinalis* infection on gut microbiota development of the puppies, as well as gut health markers of immunity such as fecal IgA and calprotectin. Sequencing results revealed that the canine fecal microbiota of *Giardia*-infected puppies becomes more complex and less diverse with increasing age. In addition, significant differences in the structure of the microbiota were observed between puppies with high and low *Giardia* cyst excretion. Chronic subclinical *G. intestinalis* infection appears to be associated with some detrimental structural changes in the gut microbiota. *G. intestinalis*-associated dysbiosis is characterized by an enrichment of facultative anaerobic, mucus-degrading, pro-inflammatory species and opportunistic pathogens, as well as a reduction of *Lactobacillus johnsonii* at specific time points. Calprotectin levels increased with age, suggesting the establishment of chronic low-grade inflammation in puppies. Further work is needed to demonstrate whether these alterations in the canine gut microbiota could lead to a dysbiosis-related disease, such as irritable bowel syndrome (IBS) or inflammatory bowel disease (IBD).

## 1. Introduction

*Giardia intestinalis* (syn. *Giardia duodenalis*, *Giardia lamblia*) is a flagellate protozoan responsible for giardiosis (or giardiasis in humans), one of the most prevalent and widespread parasitic infection in humans and mammals worldwide [1,2]. Clinical manifestations of acute giardiosis include intestinal malabsorption, diarrhea, abdominal pain, and weight loss [3]. However, *G. intestinalis* infection is often asymptomatic and chronic infections are common [4,5,6]. *G. intestinalis* is classified into eight assemblages that have different host specificities. Assemblages A and B are found in humans and other mammals, C and D in canids, E in ungulates animals, F in cats, G in rodents, and H in pinnipeds. Assemblages A and B are considered zoonotic [7]. The prevalence of *G. intestinalis* in dogs ranges from 4 to 36.5% in Europe [4,8,9]. The prevalence is higher in young animals (between 9 and 14 weeks of age, 62.4%) compared to puppies between 5 and 8 weeks of age (30.4–32.6%) [10,11,12]. Animals living in communities, for example, in shelters or breeding kennels, are more frequently positive than privately owned pets, due to the high density of animals in these facilities [4,9,13].

The gut microbiota has great diversity and it is well known that any alteration can result in an impact on health [14]. The human and canine gut microbiota are very similar in composition and share similar functions due to a long period of domestication and co-evolution of dogs with humans [15,16]. Similar to humans, the canine microbiota is established with aerobic species at birth and evolves very rapidly during the first weeks of age in favor of anaerobes [17]. In adult dogs, the microbiota is stable over the years. Similar to humans, the composition of the canine gut microbiota is affected by several factors such as diet [16], body weight [18], geographic location [19], genetics [20], age [21,22], and infections [17,23].

Nowadays, it has been known that the gut microbiota can impact the ability of *G. intestinalis* to colonize the host. Singer and Nash were the first to observe that mice with identical genetic background but from two different breedings showed different susceptibility to *G. intestinalis* infection [24]. When mice were housed together, resistance to infection was readily transferred to normally susceptible mice. Antibiotic treatment completely reversed the inability of *G. intestinalis* to infect these mice, demonstrating that the differences in susceptibility to infection are due to changes in the gut microbiota present in these mice [24]. Another study demonstrated that conventional mice were more susceptible to *G. intestinalis* infection than germ-free and gnotobiotic mice, confirming the stimulatory activity of the gut microbiota on *G. intestinalis* pathogenicity [25]. More recently, the nematode model *Caenorhabditis elegans* was used to study the effects of *G. intestinalis* on the intestinal microbiota [26]. Exposure of human commensal bacteria to *G. intestinalis* prior to administration in *C. elegans* induced nematode death, whereas the same bacteria not exposed to the parasite had no effect. In addition, the authors demonstrated that *G. intestinalis* induced the expression of virulence factors in *Escherichia coli*. These results suggest that *G. intestinalis* is capable of modifying the host’s commensal gut microbiota and possibly turn on species to opportunistic pathogens [26]. *G. intestinalis* also induces virulence of gut microbiota towards human intestinal epithelial cells in vitro [27].

Many studies on *G. intestinalis* and its impact on the gut microbiota involve experimental infections of laboratory animals. Although these studies can be useful in elucidating mechanisms of action, they generally involve laboratory-adapted strains, high infectious doses and specialized animal husbandry practices, that can affect host immunity and microbiota composition and thus lead to misinterpretations. The impact of natural *G. intestinalis* infection on the gut microbiota is poorly described. Berry et al. sequenced the gut microbiota of dogs naturally infected with one or more eukaryotic parasites and found that parasite infections are associated with significant perturbations of the microbiome and that *G. intestinalis* is associated with the greatest changes in the canine gut microbiota [5], confirming the observations of Šlapeta et al. on asymptomatic adult dogs [28].

In the present study, puppies naturally infected with *G. intestinalis* were monitored for 3 months and 16S Illumina sequencing of fecal samples from different time points was performed to assess the impact of *G. intestinalis* infection on the development of the puppies’ gut microbiota, as well as gut health markers such as fecal IgA and calprotectin.

## 2. Materials and Methods

### 2.1. Dogs

Beagle puppies from a French breeding kennel (*n* = 18; 7 males and 11 females) aged 8 to 10 weeks (mean 8.9 weeks) were included in the study. These puppies were part of a two-arm randomized study in which the dogs presented in this study served as controls. No antibiotics were administered during the experimental protocol. All animals were housed in a controlled environment of 4 dogs per box. On days of fecal collection, dogs were single housed for a maximum of 4 h to allow fecal sample collection. Body weight and fecal score were monitored weekly for the full study duration. Fecal samples were collected after spontaneous defecation at 7 time points: 8 days before the start of the study (D−8) and every 14 days after the start (D14, D28, D42, D56, D70, and D85). Fecal samples were stored at 4 and −80 °C immediately after collection until further processing.

### 2.2. Giardia Cysts Count and Genotyping

*Giardia* cysts were detected and counted by direct immunofluorescence assays (IFA) with MeriFluor^®^ *Cryptosporidium*/*Giardia* (Meridian Bioscience, Milan, Italy). Briefly, 1 g aliquots of fresh feces were diluted in 10 mL of distilled water and filtered through a sieve (mesh size: 63 µm), then IFA assays were performed in duplicate using 20 μL of this solution. Whole slides were examined under a fluorescent microscope with 20× objective (Leica Microsystems, Rueil-Malmaison, France). For genotyping, 7 mL of fecal suspension were added to 3 mL of diethylic ether, homogenized vigorously and centrifuged for 5 min at 2000× *g* at 20 °C. Pellets were washed twice with Phosphate buffered saline (PBS) and resuspended in 2 mL PBS. Samples were subjected to genomic DNA extraction using the QIAamp Mini Kit (Qiagen, Hilden, Germany), according to the manufacturer’s instructions. To disrupt parasite cyst walls, an initial step of 8 freeze-thaw cycles (freezing in liquid nitrogen for 5 min and thawing at 95 °C for 5 min) was incorporated into the protocol. Semi-nested PCR were performed to amplify glutamate dehydrogenase (*gdh*), triosephosphate isomerase (*tpi*), *β-giardin,* and *SSU* rRNA genes, as previously described [7,29,30]. The sequence of the primers is shown in Table 1. PCR products were visualized on 2% agarose gels stained with ethidium bromide (0.5 μg/mL) and amplicons were sequenced in both directions.

### 2.3. Quantification of Fecal IgA and Calprotectin

For IgA quantification, frozen fecal samples were thawed and 0.2 g aliquots of feces were diluted in 2 mL PBS. After homogenization with 0.10 to 0.25 mm diameter glass beads (Fisher Scientific) using a Precellys Evolution homogenizer (Bertin Technologies, Montigny-le-Bretonneux, France) for 40 s at 6400 rpm, suspensions were centrifuged for 5 min at 13,000× *g* at 4 °C. Supernatants were collected and stored frozen at −80 °C prior to ELISA analyses following the manufacturer’s instructions (Canine IgA, FineTest, Wuhan, China). For calprotectin quantification, fecal extracts were prepared as previously described [36]. Briefly, frozen fecal samples were thawed and 1.3 g aliquots of feces were diluted 1:5 in fecal extraction buffer (20 mM CH_3_CO_2_Na and 3 mM CaCl_2_ [pH 7.6]) containing a protease inhibitor cocktail (1 tablet/25 mL, Roche, Basel, Switzerland). After homogenization by vigorous shaking for 30 min at room temperature, the suspensions were centrifuged for 20 min at 2100× *g* at 4 °C. The supernatants were collected and centrifuged for 30 min at 10,600× *g* at 4 °C. The final supernatants (fecal extracts) were stored frozen at −80 °C prior to ELISA analyses following the manufacturer’s instructions (Canine Calprotectin, MyBiosource, San Diego, CA, USA).

### 2.4. DNA Extraction

A modified version of the protocol by Godon et al. [37] was used for DNA extraction. For each animal, 200 mg of frozen fecal samples were resuspended with a mix of 250 μL of guanidine thiocyanate buffer (4 M guanidine thiocyanate 0.1 M Tris [pH 7.5] and 40 μL of 10% N-lauroyl sarcosine 0.1 M phosphate buffer [pH 8.0]) and 500 μL of 5% N-lauroyl sarcosine, and incubated for 1 h at 70 °C. One volume (750 μL) of 0.1 mm diameter silica beads (Sigma-Aldrich, St. Louis, MI, USA) was added, and tubes were shaken for 10 min at maximum speed of a Vibrobroyeur MM200 (Retsch, Haan, Germany). Tubes were vortexed and centrifuged for 5 min at 14,000× *g* rpm at 4 °C. After recovery of the supernatant, 30 μL of Proteinase K (Chemagic STARDNA BTS kit, Perkin Elmer, Waltham, MA, USA) was added and the samples were incubated for 10 min at 70 °C at 250 rpm in Multi-Therm (Benchmark Scientific, Sayreville, NJ, USA), then for 5 min at 95 °C for enzyme inactivation. The tubes were centrifuged for 5 min at 14,000× *g* rpm at 4 °C and the supernatant was transferred to a deepwell. The plate was transferred on the nucleic acid workstation Chemagic STAR (Hamilton, Perkin Elmer, Waltham, MA, USA) and the extraction protocol was performed with Chemagic STAR DNA BTS kit (Perkin Elmer, Waltham, MA, USA) according to the manufacturer’s instructions.

### 2.5. Primer Design and Library Preparation

The V3-V4 hypervariable regions of the 16S rDNA gene were amplified from DNA extracts during the first PCR step using universal primers PCR1F_343 and PCR1_R784 (Table 1). PCR was carried out using 2 U of a DNA-free Taq DNA Polymerase and 1× Taq DNA polymerase buffer (MTP Taq DNA Polymerase, Sigma-Aldrich, St. Louis, MI, USA). The buffer was supplemented with 10 nmol of dNTP mixture (Sigma-Aldrich, St. Louis, MI, USA), 15 nmol of each primer (Eurofins, Luxembourg), and Nuclease-free water (Qiagen, Hilden, Germany) in a final volume of 50 μL. The PCR reaction was carried out in a T100 Thermal cycler (Biorad, USA) as follows: An initial denaturation step (94 °C for 10 min) was followed by 30 cycles of amplification (94 °C for 1 min, 68 °C for 1 min, and 72 °C for 1 min) and a final elongation step at 72 °C for 10 min. Amplicons were then purified using magnetic beads CleanPCR (Clean NA, GC Biotech B.V., Waddinxveen, The Netherlands) in a 96-well format. Sample multiplexing was performed by adding tailor-made 6 bp unique indexes during the second PCR step at the same time as the second part of the P5F/P7R adapters to obtain primers PCR2_P5F and PCR2_P7R (Table 1). This second PCR step was performed on 50–200 ng of purified amplicons from the first PCR using 2.5 U of a DNA free Taq DNA Polymerase and 1× Taq DNA polymerase buffer. The buffer was completed with 10 nmol of dNTP mixture (Sigma-Aldrich, St. Louis, MI, USA), 25 nmol of each primer (Eurofins, Luxembourg), and Nuclease-free water (Qiagen, Hilden, Germany) up to a final volume of 50 μL. The PCR reaction was carried out on a T100 Thermal cycler with an initial denaturation step (94 °C for 10 min), 12 cycles of amplification (94 °C for 1 min, 65 °C for 1 min, and 72 °C for 1 min) and a final elongation step at 72 °C for 10 min. Amplicons were purified as described for the first PCR reaction. All libraries were pooled with equal amounts in order to generate an equivalent number of raw reads for each library. DNA concentration of the pool was quantified on a Qubit Fluorometer (Thermo Fisher Scientific, Waltham, MA, USA). The pool at a final concentration between 5 and 20 nM was used for sequencing.

### 2.6. Illumina Sequencing

The library pool (see above) was denatured (NaOH 0.1 N) and diluted to 7 pM. Then, 15% PhiX Control v3 (Illumina, San Diego, CA, USA) was added to the pool as described in the Illumina procedure. Afterwards, 600 μL of this pool and PhiX mix were loaded onto the Illumina MiSeq cartridge according to the manufacturer’s instructions using MiSeq Reagent Kit v3 (2 × 300 bp Paired-end reads, 15 Gb output). FastQ files were generated at the end of the run (MiSeq Reporter software, Illumina, San Diego, CA, USA). The run quality was checked internally using PhiX control and each paired-end sequence was assigned to its sample using the multiplexing index. The sequences were processed using FROGS [38]. The quality of the raw sequencing data was checked using FastQC and reads with a Phred quality score <30 were discarded. Chimeras and singletons were removed from the dataset. Quality control retained sequences with a length between 100 and 400 bp. Paired-end reads were merged using Vsearch. 16S rRNA Operational Taxonomic Units (OTUs) were assigned based on at least 99% sequence similarity to the lowest possible taxonomic rank against the SILVA Pintail 100–138 reference database. One hundred percent of the sequences were affiliated. Moreover, 20.8% of sequences were multi-affiliated at the species level and 4.74% of sequences were multi-affiliated at the genus level. Biodiversity of the samples (alpha diversity) was calculated with Chao1, Shannon, and Inverse Simpson indexes, while similarity between samples (beta diversity) was calculated with Jaccard and unweighted Unifrac distances.

### 2.7. Statistical Analyses

For statistical analyses, sequences were rarefied to a uniform depth of 57,698 sequences per sample to account for unequal sequencing depth between samples. Based on rarefaction curves, samples with less than 10,000 sequence reads were excluded from the analysis. Alpha diversity indices were compared using the Mann–Whitney test (GraphPad Prism software version 9.00). The principal coordinates analysis (PCoA) was performed with FROGS to visualize the clustering of samples according to various parameters, using Jaccard and unweighted Unifrac distances. Permutational multivariate analysis of variance (PERMANOVA) was used to test for significant differences in bacterial community composition across samples. All statistical tests were two-sided, and *p* < 0.05 was considered statistically significant.

## 3. Results

### 3.1. G. intestinalis Infection Is Chronic in Puppies

**Figure 1:**
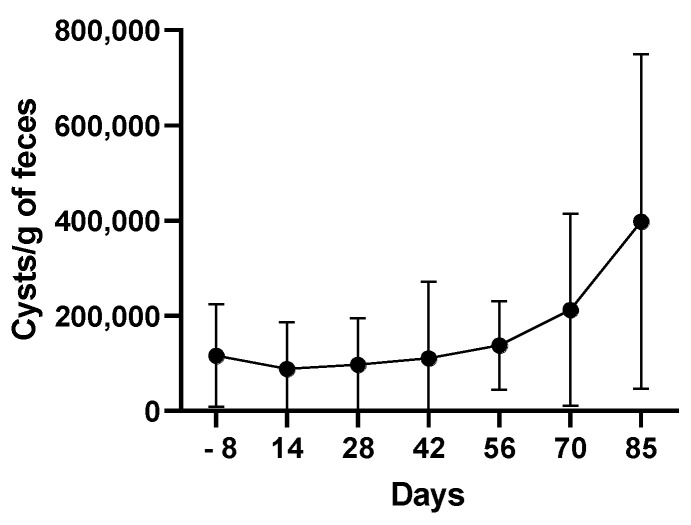
*G. intestinalis* cysts enumeration in fecal samples. Values are mean ± SEM.

Body weight and fecal score (performed using the Royal Canin Fecal Scoring Chart) were monitored weekly for 93 days and all puppies remained asymptomatic throughout the 93-day experiment (data not shown). *G. intestinalis* cysts were quantified in fecal samples at seven time points: 8 days before the start of the study (D−8) and every 14 days after the start until day 85 (D85). All animals remained infected at day 85 (Figure 1), suggesting either that puppies were not able to clear the parasite or that they were re-infected.

*G. intestinalis* genotyping may reveal different results depending on the genetic locus sequenced and multiple gene loci should be investigated to increase the specificity of the results [39]. Thus, we determined *G. intestinalis* assemblages in samples D−8 and D85 by sequencing *gdh*, *tpi*, *β-giardin*, and *SSU rRNA* genes (Table 2). All animals were infected by dog-specific assemblages C and/or D. The majority of puppies have mixed infections between assemblages in both D−8 and D85. No zoonotic A or B assemblages were detected in this study.

### 3.2. Age Influences Fecal IgA and Calprotectin Levels

**Figure 2:**
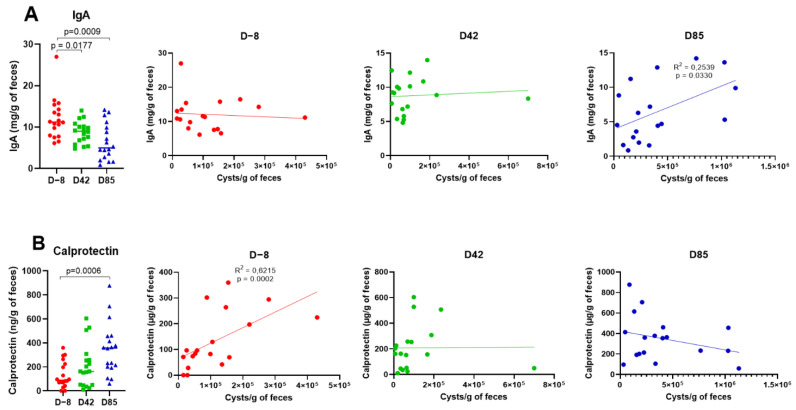
Fecal IgA (**A**) and calprotectin (**B**) concentrations and correlation with *G. intestinalis* cysts excretion in samples D−8 (red), D42 (green), and D85 (blue). Each dot represents a sample. *p*-values were determined using the Mann-Whitney test. Correlations were determined by Pearson’s coefficient.

Further analyses focused on three time points: D−8, D42, and D85 to investigate the effects of age on IgA levels and calprotectin concentrations, as non-invasive markers of intestinal health. Fecal IgA levels were measured by ELISA. As shown in Figure 2A, fecal IgA concentrations were significantly lower in D42 and D85 samples compared to the D−8 samples (*p* = 0.0177 and *p* = 0.0009, respectively). Next, we investigated whether IgA variations could be related to *G. intestinalis* cysts shedding. Fecal IgA concentrations were positively correlated with *G. intestinalis* cysts shedding in D85 samples (*R*^2^ = 0.2539; *p* = 0.0330), whereas no significant correlation was observed in D−8 and D42 samples. Fecal calprotectin levels were also measured by ELISA. As shown in Figure 2B, a significant increase in fecal calprotectin was observed in D85 samples compared to D−8 samples (*p* = 0.0006). Fecal calprotectin concentrations correlate positively with *G. intestinalis* cysts shedding in D−8 samples (*R*^2^ = 0.6215; *p* = 0.0002).

### 3.3. Age Influences the Richness and Diversity of the Canine Gut Microbiota

**Figure 3:**
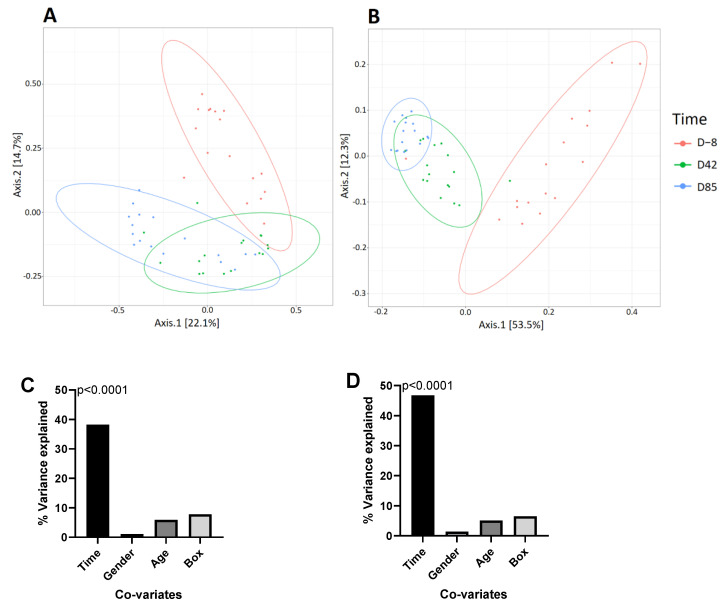
Beta diversity. Two-dimensional representation of PCoA plots based on (**A**) Jaccard and (**B**) unweighted Unifrac beta diversity of 16S rDNA genes. Each dot represents a sample, and each color represents a collection time: D−8 (red), D42 (green), and D85 (blue) samples. Axis 1 is the principal coordinate component causing the largest difference in samples, with an explanatory value of 22.1% for Jaccard and 53.5% for Unifrac. Axis 2 is next, with an explanatory value of 14.7% for Jaccard and 12.3% for Unifrac. The percent of variance in (**C**) Jaccard and (**D**) unweighted UniFrac beta diversity explained by each variable. *p*-values were determined with PERMANOVA.

To characterize the evolution of the canine gut microbiota over time in naturally-infected puppies, we used the Illumina MiSeq technology to sequence the V3-V4 region of 16S rDNA obtained from a total of 54 fecal samples (18 puppies at three different time points). Sequencing generated a total of 5.90 million reads with an average of 109,300 reads per sample. The quality and chimera filtering produced a total of 4,475,733 reads with an average of 82,883 filtered reads per sample. Beta diversity was analyzed based on Jaccard and unweighted Unifrac distance matrices and represented by PCoA. The predicted PCoA representation of Jaccard and unweighted Unifrac beta diversity (Figure 3) and sample clustering by Ward D2 (Figure S1) revealed separate clusters for samples D−8, D42, and D85. The variable “collection time” explained 38.20% of the variance using Jaccard distance (PERMANOVA, *p* < 0.0001) and 46.71% of the variance using Unifrac distance (PERMANOVA, *p* < 0.0001). PERMANOVA was also applied to assess the similarities of microbial communities as a function of gender, age, and box in both distance matrices. None of these other variables were significantly associated with microbiota composition (gender: *p* = 0.4453; age: *p* = 0.2073; box: *p* = 0.0535), which is consistent with previous studies [13,20].

**Figure 4:**
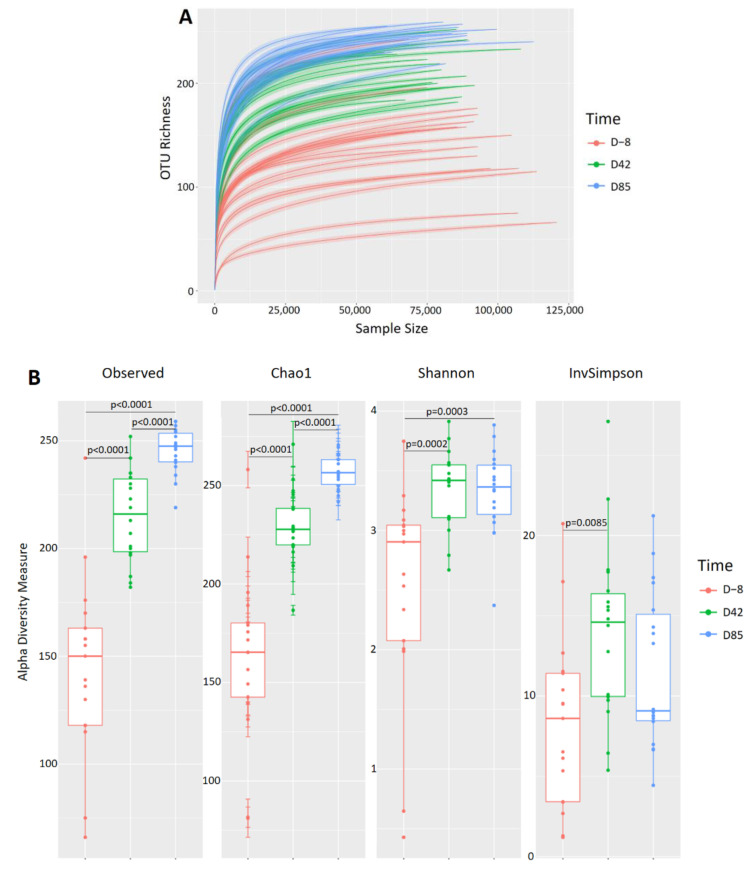
(**A**) Rarefaction curves, (**B**) alpha diversity measures. *p*-values were determined using the Mann–Whitney test.

Rarefaction curves generated with these reads revealed that the sequencing depth appropriately covered bacterial diversity in all samples (Figure 4A). Samples collected in D−8 had a lower level of complexity, with an average of 146 ± 43 OTUs, compared to D42 and D85 samples, which had an average of 218 ± 21 OTUs and 251 ± 11 OTUs, respectively. Observed OTUs, Chao1, Shannon, and Inverse Simpson richness were used to determine taxonomic diversity within samples (Figure 4B). All diversity measures were significantly increased in D42 samples compared to D−8 samples (ANOVA *p* < 0.0001; *p* < 0.0001; *p* = 0.0002; *p* = 0.0085, respectively). In D85 samples, observed OTUs and Chao1 alpha diversity increase compared to D42 samples (ANOVA *p* < 0.0001), while no significant differences were observed for Shannon and Inverse Simpson measures, suggesting that microbiota richness tends to stabilize over time in an adult-like microbiota.

**Figure 5:**
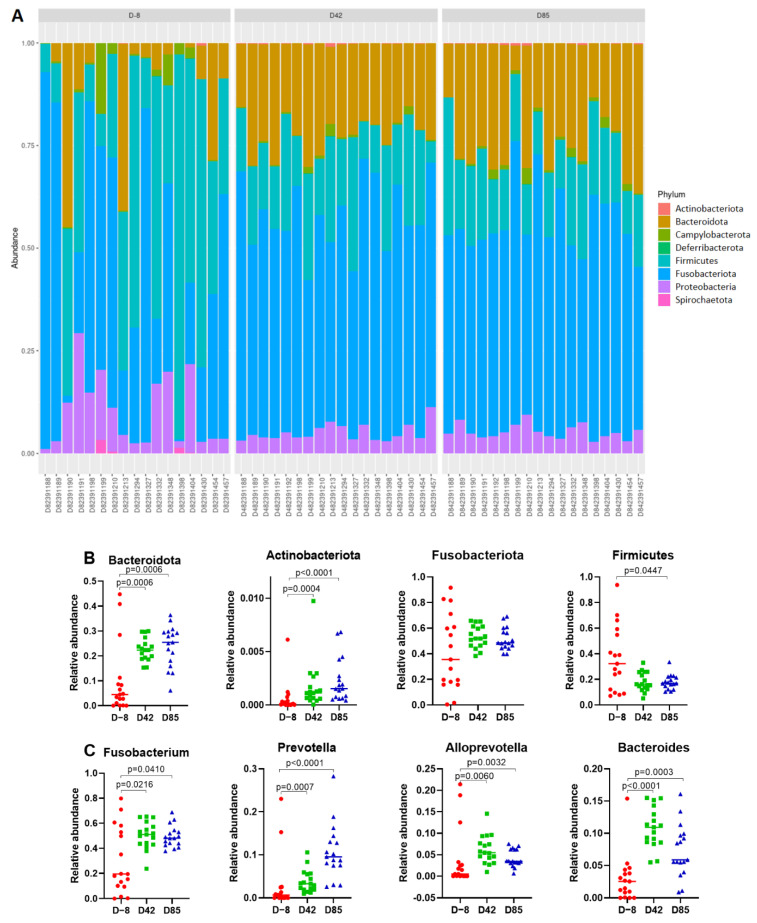

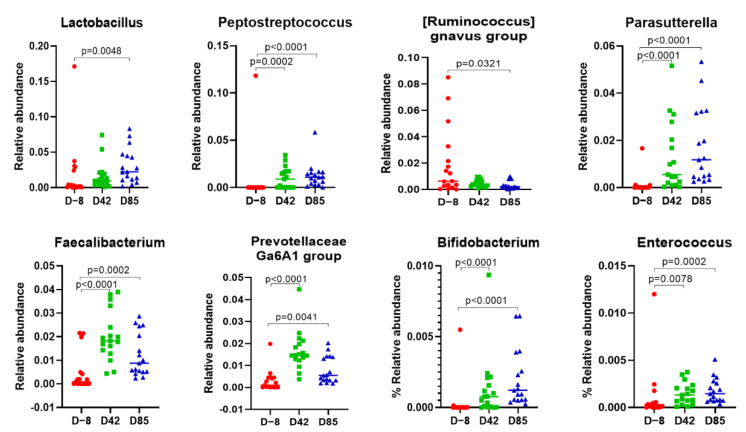
(**A**) Relative abundance of the eight phyla in fecal samples at D−8 (red), D42 (green), and D85 (blue). Differentially abundant phyla (**B**) and genera (**C**) among samples D−8, D42, and D85. Each dot represents a sample. *p*-values were determined using the Mann-Whitney test.

A total of eight phyla and 81 genera were identified (Figure 5A). High inter-individual variability was observed in puppies at D−8 and decreased in older puppies at D42 and D85. Despite this high variability, core bacterial groups were identified. At the phylum level, the core microbiota was dominated by taxa belonging to Fusobacteriota (48.7 ± 18.9%), Firmicutes (24.1 ± 17.6%), Bacteriodota (18.8 ± 11.6%), and Proteobacteria (6.8 ± 5.6%). The minor abundant members are phyla Campylobacterota (1.3 ± 2.6%), Spirochaetota (0.1 ± 0.4%), Actinobacteriota (0.2 ± 0.2%), and Deferribacterota (2 × 10^−4^ ± 2 × 10^−4^%). The abundance of the four dominant phyla varies greatly with time. Bacteroidota and Actinobacteriota significantly increase in D42 samples (*p* = 0.0006 and *p* = 0.0004, respectively) and in D85 samples (*p* = 0.0006 and *p* > 0.0001, respectively) compared to the D−8 samples. In contrast, Firmicutes abundance decreased in D85 samples compared to the D−8 samples (*p* = 0.0447). Fusobacteriota abundance is highly variable in D−8 samples, and tends to stabilise with increasing age in D42 and D85 samples. At the genus level, the abundance of most dominant genera was significantly enriched in D85 samples compared to the D−8 samples, including *Fusobacterium* (*p* = 0.0410), *Prevotella* (*p* < 0.0001), *Alloprevotella* (*p* = 0.0032), *Lactobacillus* (*p* = 0.0048), *Bacteroides* (*p* = 0.0003), *Peptostreptococcus* (*p* < 0.0001), *Ruminococcus gnavus* group (*p* = 0.0321), *Parasuterella* (*p* < 0.0001), *Faecalibacterium* (*p* = 0.0002), *Prevotella* Ga6A1 group (*p* < 0.0001), *Bifidobacterium* (*p* < 0.0001), and *Enterococcus* (*p* = 0.0002).

**Figure 6:**
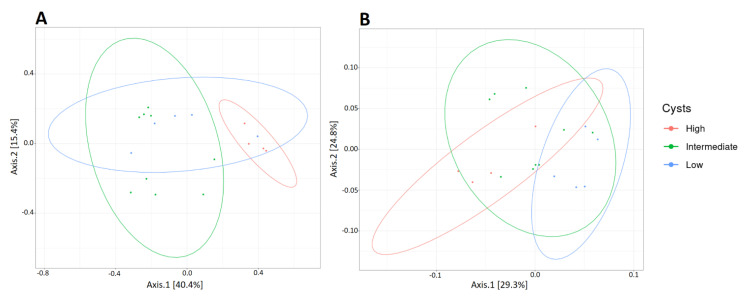
Two-dimensional representation of PCoA plots based on (**A**) Jaccard and (**B**) unweighted Unifrac beta diversity of 16S rDNA genes. Each dot represents a sample, and each color represents a collection time: D−8 (red), D42 (green), and D85 (blue). Axis 1 is the principal coordinate component causing the largest difference in samples, with an explanatory value of 40.4% for Jaccard and 29.3% for Unifrac. Axis 2 was next, with an explanatory value of 15.4% for Jaccard and 24.8% for Unifrac.

*G. intestinalis* affects the richness and diversity of the canine gut microbiota. To determine the impact of *G. intestinalis* on the composition of the fecal microbiota, we classified the samples into three categories according to their parasite load: Low, intermediate, and high. The beta diversity of these three categories was then analyzed using Jaccard and unweighted Unifrac distance matrices and was represented through PCoA (Figure 6). In samples D−8 and D42, no difference in the structure of the overall microbiota as a function of parasitic load was observed (data not shown). However, in D85 samples, when the parasite load spectrum is broader, the samples cluster significantly with the parasite load (low <150,000 cysts/g of feces, intermediate between 150,000 and 750,000 cysts/g of feces, and high >750,000 cysts/g of feces). The parasite load explains 19.18% of the variance using the Jaccard distance (PERMANOVA *p* = 0.0404) and 23.18% of the variance using unweighted Unifrac distance (PERMANOVA *p* = 0.0152).

**Figure 7:**
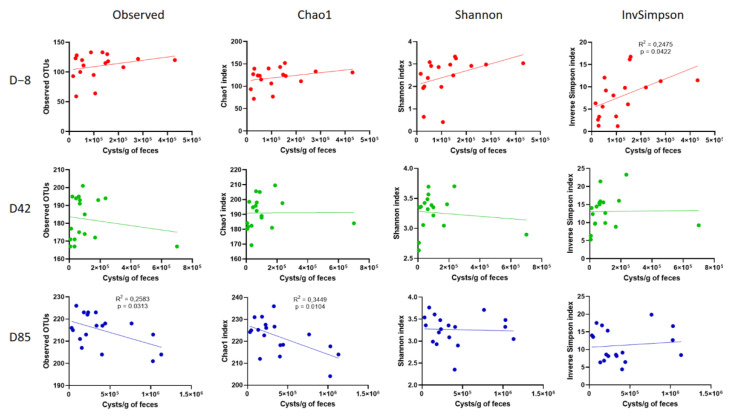
Correlation between alpha diversity measures and *G. intestinalis* cysts shedding in D−8 samples (red), D42 samples (green), and D85 samples (blue). The correlation was determined using Pearson’s coefficient.

In addition, a significant correlation was observed between alpha diversity and *G. intestinalis* cysts excretion at the three time points (Figure 7). Bacterial richness depends on the age of the animal. On D−8, Inverse Simpson richness correlates positively with cysts excretion in young puppies (*R*^2^ = 0.2475; *p* = 0.0422). A positive trend was also noted in the observed, Chao1, and Shannon richness but without reaching the significance threshold of 0.05. On the contrary, when puppies are older and have a more mature microbiota, cysts excretion correlates with a significant decrease in the observed and Chao1 richness (*R*^2^ = 0.2583; *p* = 0.0313 and *R*^2^ = 0.3449; *p* = 0.0104, respectively). At D42, a slight decrease in the observed richness was noted but no significant richness difference, which is consistent with the intermediate stage.

**Figure 8:**
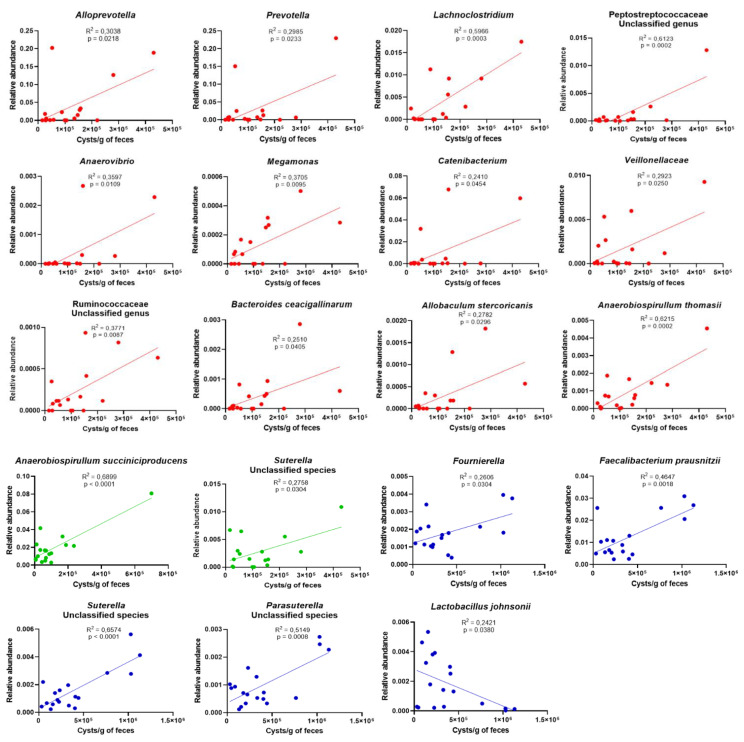
Correlation between cysts shedding and OTU relative abundance in samples D−8 (red), D42 (green), and D85 (blue). The correlation was determined using Pearson’s coefficient.

The abundance of specific OTUs correlates significantly with *G. intestinalis* cysts shedding at the three time points (Figure 8). In D−8 samples, *G. intestinalis* cysts shedding correlates positively with *Alloprevotella* (*R*^2^ = 0.3038; *p* = 0.0218), *Prevotella* (*R*^2^ = 0.2985; *p* = 0.0233), *Lachnoclostridium* (*R*^2^ = 0.5966; *p* = 0.0003), an unclassified genus of *Peptostreptococcaceae* (*R*^2^ = 0.6123; *p* = 0.0002), *Anaerovibrio* (*R*^2^ = 0.3597; *p* = 0.0109), *Megamonas* (*R*^2^ = 0.3705; *p* = 0.0095), *Catenibacterium* (*R*^2^ = 0.2410; *p* = 0.454), *Veillonellaceae* (*R*^2^ = 0.2923; *p* = 0.0250), an unclassified genus of *Ruminococcaceae* (*R*^2^ = 0.3771; *p* = 0.0087), *Bacteroides caecigallinarum* (*R*^2^ = 0.2510; *p* = 0.0405), *Allobaculum stercoricanis* (*R*^2^ = 0.2782; *p* = 0.0296), and *Anaerobiospirullum thomasii* (*R*^2^ = 0.6215; *p* = 0.0002). In D42 samples, *G. intestinalis* cysts shedding correlates positively with *Anaerobiospillum succiniproducens* (*R*^2^ = 0.6899; *p* < 0.0001) and an unclassified species of *Suterella* (*R*^2^ = 0.2758; *p* = 0.0304). In D85 samples, *G. intestinalis* cysts shedding correlates positively with *Fournierella* (*R*^2^ = 0.2606; *p* = 0.0304), *Faecalibacterium prausnitzii* (*R*^2^ = 0.4647; *p* = 0.0018), and unclassified species of *Suterella* (*R*^2^ = 0.6574; *p* < 0.0001) and *Parasuterella* (*R*^2^ = 0.5149; *p* = 0.0008). On the contrary, *G. intestinalis* cysts shedding correlates negatively with *Lactobacillus johnsonii* in D85 samples (*R*^2^ = 0.2421; *p* = 0.0380).

## 4. Discussion

*G. intestinalis* is one of the most common enteric parasites worldwide and is remarkable for its ability to cause an array of clinical phenotypes, ranging from asymptomatic infections to severe acute diarrheal disease to chronic gastrointestinal disease in human and animals. Acute giardiasis is auto-resolutive in 2 to 4 weeks in adults but could become chronic especially in young subjects, resulting in serious growth stunting and long-term health consequences [40,41]. In this study, 18 puppies (9 weeks of age) naturally infected with *G. intestinalis* remained infected at 21 weeks of age (D85) when no rescue treatment was administered, suggesting that *G. intestinalis* infection was not auto-resolutive in these animal hosts. In a murine model of infection, animals infected neonatally did not clear the parasite when they reached adulthood, although naive adult animals exposed to *G. intestinalis* were resistant to infection, suggesting that *G. intestinalis* manipulated the host during the postnatal period to favor its own persistence [42]. Genotyping results revealed that puppies were infected by assemblages C and D, and that multi-infections were frequent, confirming previous observations [12,43,44].

IgA is the predominant Ig subtype present in host secretions and protects mucosal surface from infectious agents. IgA plays a role in *G. intestinalis* clearance by targeting the parasite variant-specific surface proteins (VSPs) [45,46]. We demonstrated a decrease of fecal IgA concentration with age, which has been described elsewhere and is explained by the loss of IgA provided by maternal milk after weaning [11,47,48]. Fecal IgA correlated positively with *G. intestinalis* cysts shedding at day 85. No correlation was observed in earlier time points (Figure 2A). These results are consistent with a previous study in *G. muris* infected mice where secretions of total and parasite-specific IgA were observed after weaning. This study demonstrated that a protective intestinal immune response started to develop only after weaning, which could explain the prolonged course of infection in neonate mice in comparison to older mice [49]. However, in our study, the IgA immune response at D85 was not sufficient to eradicate the *G. intestinalis* infection in weaned puppies.

Calprotectin is a protein complex mostly abundant in neutrophiles, monocytes, and reactive macrophages at sites of inflammation. Since it reflects the phagocyte turnover in vivo, calprotectin has been used as a marker of inflammation that correlates with local and systemic signs of disease activity [50,51]. Previous studies have observed a reduction of fecal calprotectin levels with age in healthy puppies [52] and children [53]. In the current study, fecal calprotectin levels increased with age in puppies naturally infected with *G. intestinalis*. Fecal calprotectin concentration was associated with *G. intestinalis* cysts shedding in 9-week-old (D−8) puppies. No correlation was observed in later time points (Figure 2B), in accordance with earlier findings in puppies [11] and asymptomatic children [53,54]. On the contrary, levels of fecal calprotectin are elevated in infected patients presenting clinical signs [55]. Here, all puppies remained asymptomatic during the course of the study, explaining the lack of correlation between cysts shedding and calprotectin levels in later time points.

The canine fecal microbiota was investigated during 12 weeks and revealed a clear gradual shift from a simple and micro-aerobic microbiota in 9-week-old puppies (D−8) to a more complex, homogeneous and anaerobic microbiota in 21-week-old puppies (D85) (Figure 4). An increased richness with age has already been described in dogs [17] and humans [56]. At 21 weeks of age (D85), puppies present an adult-like microbiota dominated by four phyla: Bacteroidota, Firmicutes, Fusobacteriota, and Proteobacteria (Figure 5A). Although the predominant phyla identified are similar to previous observations in healthy dogs, proportions vary among studies [57,58,59]. The canine breed, environmental conditions, age, weight, infections, sequencing technologies, and sequencing depth are responsible for significant variations reported for gut microbiota composition [18,19,20,21,22,23]. Here, there were more pronounced differences in the composition of fecal microbiota among 9-week-old puppies (D−8) compared to 21-week-old puppies (D85). This high inter-individual variability could be explained by the fact that some puppies are already weaned for a couple of weeks, while others just finished the weaning process. After weaning, the composition of the gut microbiota in puppies seems to vary greatly (Figure 5B,C), notably by increasing *Bacteroides* and *Prevotella*, which is already well described in dogs [17,60] and humans [61,62]. Other genera are enriched after weaning, including *Fusobacterium*, *Alloprevotella*, *Peptostreptococcus*, [*Ruminoccocus*] *gnavus* group, *Parasuterella*, *Faecalibacterium*, and Prevotellaceae Ga6A1 group. Many of these microbes are efficient degraders of dietary fibers and producers of short chain fatty acids (SCFAs). SCFAs display health properties that promote host intestinal health. For example, they regulate cell differentiation and proliferation, gut endocrine functions, immune response, and promote the intestinal epithelial barrier function. Finally, butyrate (one of the main SCFAs) is the primary source of energy for enterocytes [63,64]. The consumption of a solid diet, which in turn may modify the substrate availability and the physiological conditions of the gastrointestinal tract, was probably the main cause associated with the increased functional capacity for carbohydrates degradation. Surprisingly, genera *Bifidobacterium*, *Lactobacillus*, and *Enterococcus* increased with age, contrasting with findings in humans [65].

Most research of *Giardia* impact on the gut microbiota focused on animals experimentally infected with laboratory adapted strains and little is known about the canine microbiota during natural infections, in particular in asymptomatic animals. Some report described altered composition [5,28], while others showed modest or no effect in adult dogs [66]. These studies compared *Giardia*-positive to *Giardia*-negative individuals. In this study, all animals are infected by the parasite and the effects of the parasite load on the fecal microbiota composition were investigated instead. Results showed that the gut microbiota structure was dependent on the parasite load, as puppies with high cysts shedding displayed altered microbiota compared to puppies with a low cyst load (Figure 6). Furthermore, *G. intestinalis* impact on microbiota richness seemed to be related to the age of the host. In 9-week-old puppies with immature gut microbiota (D−8), *G. intestinalis* was associated with increased bacterial richness, while in older animals with adult-like gut microbiota (D85), the parasite load correlated positively with decreased bacterial richness (Figure 7). These results are similar to previous findings in asymptomatic children [67] and mice [68]. In either case, an alteration of microbial richness was found to be detrimental for the host. Increased richness in young subjects and lower microbial diversity in adults are often associated with a disease state [69,70].

*G. intestinalis* correlates positively with pro-inflammatory *Prevotella*, *Suterella*, Veillonellaceae, and an unclassified genus of Ruminococcaceae (Figure 8). *Prevotella* is a major component of the gut microbiota of mammals and generally associated with gut health [71]. However, when compared with strict commensal bacteria, *Prevotella* exhibits increased inflammatory properties and might participate in disease by promoting chronic inflammation [72]. Moreover, perturbation of the gut microbiome by *Prevotella* enhances host susceptibility to mucosal inflammation [73]. Increased levels of *Prevotella* during *G. intestinalis* infection have already been reported in adult dogs and children with and without clinical signs [5,67,74,75]. This enrichment of pro-inflammatory species might be responsible for the increased level of fecal calprotectin observed in this study (Figure 2B), while it normally tends to decrease with age in healthy puppies [52].

In addition, inflammation impairs the ability of the intestinal epithelium to perform β-oxidation leading to oxygen diffusion in the gut lumen thus promoting colonization with facultative anaerobic Proteobacteria and enteric pathogens, as observed in this study [76,77,78,79]. Indeed, *G. intestinalis* correlates positively with facultative anaerobic Peptostreptococcaceae, Veillonellaceae, and members of Proteobacteria including *Suterella*, *Parasuterella*, *A. succiniproducens*, and *A. thomasii* (Figure 8), as reported elsewhere [5,80]. *Giardia* itself could also be responsible for intestinal epithelium disruption due to its ability to induce enterocyte apoptosis and tight junction destabilization [81,82].

*G. intestinalis* infection is associated with an enrichment of potentially harmful bacteria (Figure 8) such as *Prevotella* and *A. succiniproducens* that have been reported as opportunistic pathogens [83,84,85] and genera *Megamonas* and *Catenibacterium*, associated with abdominal pain and aggressive behavioral disorder in dogs [86,87].

Mucus layer plays an important role in protecting intestinal epithelial cells from commensal gut microbiota and pathogenic microorganisms. Mucin inhibited *G. intestinalis* adhesion to intestinal epithelial cells [88]. In this study, *G. intestinalis* positively correlates with Peptostreptococcaceae (Figure 8), that is significantly associated with reduction of MUC2 expression and downregulation of tight junction proteins expression [89]. *G. intestinalis* also positively correlates with *Prevotella* and *Bacteroides* (Figure 8), both genera containing members able to degrade host mucins [5,90,91]. These mucolytic bacteria could act in synergy with *G. intestinalis* cysteine proteases and degrade the protective mucus layer [92], promoting both parasite colonization and invasion of commensal bacteria [93,94,95]. These changes may enable easy contact of microbes with epithelial cells, stimulating an inflammatory response.

Epidemiological studies have linked *G. intestinalis* outbreaks to important post-infectious complications in humans, especially the development of irritable bowel syndrome (IBS) after the clearance of the parasite [41,93,96]. In addition, dogs are able to develop an IBS-like condition, similar to that described in humans [97]. Finally, in other studies, most of bacterial members that correlate with *G. intestinalis* infection in puppies are known to be associated with inflammatory bowel disease (IBD), including families Ruminococcaceae [98], Peptostreptococcaceae [99,100], and Veillonellaceae [101,102,103] and genera *Prevotella* [104], *Alloprevotella* [105,106,107], *Lachnoclostridium* [107], *Suterella* [99,108], *Parasuterella* [109,110], and *Bacteroides* [111,112,113]. Altogether, these results suggest that dysbiosis associated with chronic *G. intestinalis* infection might predispose puppies to later development of either post-infectious IBS or IBD similarly to humans.

*L. johnsonii* correlates negatively with *G. intestinalis* in puppies at D85 (Figure 8). *L. johnsonii* is the most common species among the isolates from the pre-weaning dogs, suggesting that *L. johnsonii* might be a specific species of infant dogs [60]. *L. johnsonii* species is known for its probiotic properties and probably plays an important role in the development of a healthy gut in puppies. Allain et al. have demonstrated that *L. johnsonii* La1 possess anti-giardial activity through the production of bile salt hydrolases in vitro and in vivo in a murine model of infection [114]. Thus, probiotics represent a promising alternative strategy for the control of *G. intestinalis* infection [115].

## 5. Conclusions

The aim of this study was to describe the gut microbiota of growing puppies naturally infected with *G. intestinalis*. 16S Illumina sequencing revealed that the canine fecal microbiota becomes more complex and less diverse with age. Although some studies have linked the presence of intestinal parasites to gut health [116,117], subclinical chronic *G. intestinalis* infection in puppies appears to induce some detrimental structural changes in the gut microbiota. *G. intestinalis*-associated dysbiosis is characterized by an enrichment of facultative anaerobic, mucus-degrading, pro-inflammatory species, and opportunistic pathogens, as well as in a reduction of *L. johnsonii* at specific time points. These results do not resolve whether infection is responsible for these changes. It is possible that certain compositions of the gut microbiota confer susceptibility or resistance to colonization by the parasite, as has been suggested before [24,25]. On the other hand, *G. intestinalis* infection is associated with electrolyte, water and nutrient malabsorption, arginine deprivation, brush-border microvilli shortening, and disaccharidase deficiencies [118]. By modulating the availability of resources for commensal bacteria, these pathological changes could explain the alterations in the gut microbiota observed during *G. intestinalis* infection. Finally, the increased calprotectin levels illustrate the establishment of a chronic low-grade inflammation in puppies. However, further work is needed to demonstrate whether these alterations in the canine gut microbiota could lead to a dysbiosis-related disease, such as IBS or IBD.

## Figures and Tables

**Table 1:** Primers used in this study. *Gdh*, *tpi*, *B-giardin,* and *SSU rRNA* target *Giardia*, V3-V4 *16S rDNA* target the microbiota.

Target	Name	Sequence	Reference
*gdh*	GDHeF	TCAACGTYAAYCGYGGYTTCCGT	[29]
	GDHiF	CAGTACAACTCYGCTCTCGG	
	GDHiR	GTTRTCCTTGCACATCTCC	
*tpi*	AL3543	AAATIATGCCTGCTCGTCG	[30]
	AL3544	CCCTTCATCGGIGGTAACTT	
	AL3545	GTGGCCACCACICCCGTGCC	
	AL3546	CAAACCTTITCCGCAAACC	
*β-giardin*	G7	AAGCCCGACGACCTCACCCGCAGTGC	[31]
	G759	GAGGCCGCCCTGGATCTTCGAGACGAC	
	G99	GAACGAACGAGATCGAGGTCCG	[32]
	G609	CTCGACGAGCTTCGTGTT	
*SSU rRNA*	RH11	CATCCGGTCGATCCTGCC	[33]
	RH4	AGTCGAAC CCTGATTCTCCGCCCAGG	
	GIAR-F	GACGCTCTCCCCAAGGAC	[29]
	GIAR-R	CTGCGTCACGCTCG	
V3-V4 *16S rDNA*	PCR1F_343	CTTTCCCTACACGACGCTCTTCCGATCT- ACGGRAGGCAGCAG partial P5 adapter–primer	[34]
	PCR1_R784	GGAGTTCAGACGTGTGCTCT- TCCGATCTTACCAGGGTATCTAATCCT partial P7 adapter–primer	
	PCR2_P5F	AATGATACGGCGACCACCGAGATCTACACT-CTTTCCCTACACGAC partial P5 adapter–primer targeting primer 1F	[35]
	PCR2_P7R	CAAGCAGAAGACGGCATACGAGAT-NNNNNN-GTGACT-GGAGTTCAGACGTGT partial P7 adapter including index–primer targeting primer 1R	

**Table 2:** Number of *G. intestinalis* assemblages sequenced at *gdh*, *tpi*, *β-giardin,* and *SSU rRNA* loci.

	Gene Loci			
	*gdh*	*tpi*	*β-giardin*	*SSU rRNA*
D−8	Assemblage C = 6 Assemblage D = 11 Assemblage C or D = 0 Not amplified = 1	Assemblage C = 13 Assemblage D = 2 Assemblage C or D = 0 Not amplified = 3	Assemblage C = 2 Assemblage D = 14 Assemblage C or D = 0 Not amplified = 2	Assemblage C = 0 Assemblage D = 2 Assemblage C or D = 14 Not amplified = 2
D85	Assemblage C = 7 Assemblage D = 2 Assemblage C or D = 0 Not amplified = 9	Assemblage C = 7 Assemblage D = 0 Assemblage C or D = 0 Not amplified = 11	Assemblage C = 4 Assemblage D = 1 Assemblage C or D = 0 Not amplified = 13	Assemblage C = 3 Assemblage D = 5 Assemblage C or D = 6 Not amplified = 4

## Data Availability

Sequencing data analyzed here are publicly available on the Sequence Read Archive (SRA) under study accession number PRJNA759134.

## Supplementary Materials

The following are available online at https://www.mdpi.com/article/10.3390/microorganisms9091862/s1, Figure S1: Beta diversity. Sample clustering using (A) Jaccard or (B) unweight Unifrac beta diversity.

## References

[1] Cernikova L., Faso C., Hehl A.B. (2018). Five Facts about Giardia Lamblia. PLoS Pathog..

[2] Santin M. (2020). Cryptosporidium and Giardia in Ruminants. Vet. Clin. N. Am. Food Anim. Pract..

[3] Buret A.G., Cacciò S.M., Favennec L., Svärd S. (2020). Update on *Giardia*: Highlights from the Seventh International *Giardia* and *Cryptosporidium* Conference. Parasite.

[4] Adell-Aledón M., Köster P.C., de Lucio A., Puente P., Hernández-de-Mingo M., Sánchez-Thevenet P., Dea-Ayuela M.A., Carmena D. (2018). Occurrence and Molecular Epidemiology of Giardia Duodenalis Infection in Dog Populations in Eastern Spain. BMC Vet. Res..

[5] Berry A.S.F., Johnson K., Martins R., Sullivan M.C., Farias Amorim C., Putre A., Scott A., Wang S., Lindsay B., Baldassano R.N. (2020). Natural Infection with *Giardia* Is Associated with Altered Community Structure of the Human and Canine Gut Microbiome. mSphere.

[6] Tysnes K.R., Skancke E., Robertson L.J. (2014). Subclinical Giardia in Dogs: A Veterinary Conundrum Relevant to Human Infection. Trends Parasitol..

[7] Cacciò S.M., Lalle M., Svärd S.G. (2018). Host Specificity in the Giardia Duodenalis Species Complex. Infect. Genet. Evol..

[8] Hinney B., Gottwald M., Moser J., Reicher B., Schäfer B.J., Schaper R., Joachim A., Künzel F. (2017). Examination of Anonymous Canine Faecal Samples Provides Data on Endoparasite Prevalence Rates in Dogs for Comparative Studies. Vet. Parasitol..

[9] Uiterwijk M., Nijsse R., Kooyman F.N.J., Wagenaar J.A., Mughini-Gras L., Ploeger H.W. (2019). Host Factors Associated with Giardia Duodenalis Infection in Dogs across Multiple Diagnostic Tests. Parasit. Vectors.

[10] Bouzid M., Halai K., Jeffreys D., Hunter P.R. (2015). The Prevalence of Giardia Infection in Dogs and Cats, a Systematic Review and Meta-Analysis of Prevalence Studies from Stool Samples. Vet. Parasitol..

[11] Grellet A., Heilmann R.M., Polack B., Feugier A., Boucraut-Baralon C., Grandjean D., Grützner N., Suchodolski J.S., Steiner J.M., Chastant-Maillard S. (2016). Influence of Breed Size, Age, Fecal Quality, and Enteropathogen Shedding on Fecal Calprotectin and Immunoglobulin A Concentrations in Puppies During the Weaning Period. J. Vet. Intern. Med..

[12] Sommer M.F., Rupp P., Pietsch M., Kaspar A., Beelitz P. (2018). Giardia in a Selected Population of Dogs and Cats in Germany—Diagnostics, Coinfections and Assemblages. Vet. Parasitol..

[13] Liao S., Lin X., Sun Y., Qi N., Lv M., Wu C., Li J., Hu J., Yu L., Cai H. (2020). Occurrence and Genotypes of *Cryptosporidium* spp., *Giardia duodenalis*, and *Blastocystis* sp. in Household, Shelter, Breeding, and Pet Market Dogs in Guangzhou, Southern China. Sci. Rep..

[14] Minamoto Y., Otoni C.C., Steelman S.M., Büyükleblebici O., Steiner J.M., Jergens A.E., Suchodolski J.S. (2015). Alteration of the Fecal Microbiota and Serum Metabolite Profiles in Dogs with Idiopathic Inflammatory Bowel Disease. Gut Microbes.

[15] Alessandri G., Milani C., Mancabelli L., Mangifesta M., Lugli G.A., Viappiani A., Duranti S., Turroni F., Ossiprandi M.C., van Sinderen D. (2019). Metagenomic Dissection of the Canine Gut Microbiota: Insights into Taxonomic, Metabolic and Nutritional Features. Environ. Microbiol..

[16] Coelho L.P., Kultima J.R., Costea P.I., Fournier C., Pan Y., Czarnecki-Maulden G., Hayward M.R., Forslund S.K., Schmidt T.S.B., Descombes P. (2018). Similarity of the Dog and Human Gut Microbiomes in Gene Content and Response to Diet. Microbiome.

[17] Guard B.C., Mila H., Steiner J.M., Mariani C., Suchodolski J.S., Chastant-Maillard S. (2017). Characterization of the Fecal Microbiome during Neonatal and Early Pediatric Development in Puppies. PLoS ONE.

[18] Chun J.L., Ji S.Y., Lee S.D., Lee Y.K., Kim B., Kim K.H. (2020). Difference of Gut Microbiota Composition Based on the Body Condition Scores in Dogs. J. Anim. Sci. Technol..

[19] Jha A.R., Shmalberg J., Tanprasertsuk J., Perry L., Massey D., Honaker R.W. (2020). Characterization of Gut Microbiomes of Household Pets in the United States Using a Direct-to-Consumer Approach. PLoS ONE.

[20] Reddy K.E., Kim H.-R., Jeong J.Y., So K.-M., Lee S., Ji S.Y., Kim M., Lee H.-J., Lee S., Kim K.-H. (2019). Impact of Breed on the Fecal Microbiome of Dogs under the Same Dietary Condition. J. Microbiol. Biotechnol..

[21] Kubinyi E., Bel Rhali S., Sándor S., Szabó A., Felföldi T. (2020). Gut Microbiome Composition Is Associated with Age and Memory Performance in Pet Dogs. Animals.

[22] Mizukami K., Uchiyama J., Igarashi H., Murakami H., Osumi T., Shima A., Ishiahra G., Nasukawa T., Une Y., Sakaguchi M. (2019). Age-Related Analysis of the Gut Microbiome in a Purebred Dog Colony. FEMS Microbiol. Lett..

[23] Park J.S., Guevarra R.B., Kim B.-R., Lee J.H., Lee S.H., Cho J.H., Kim H., Cho J.H., Song M., Lee J.-H. (2019). Intestinal Microbial Dysbiosis in Beagles Naturally Infected with Canine Parvovirus. J. Microbiol. Biotechnol..

[24] Singer S.M., Nash T.E. (2000). The Role of Normal Flora in *Giardia Lamblia* Infections in Mice. J. Infect. Dis..

[25] Torres M.F., Uetanabaro A.P.T., Costa A.F., Alves C.A., Farias L.M., Bambirra E.A., Penna F.J., Vieira E.C., Nicoli J.R. (2000). Influence of Bacteria from the Duodenal Microbiota of Patients with Symptomatic Giardiasis on the Pathogenicity of Giardia Duodenalis in Gnotoxenic Mice. J. Med. Microbiol..

[26] Gerbaba T.K., Gupta P., Rioux K., Hansen D., Buret A.G. (2015). *Giardia Duodenalis*-Induced Alterations of Commensal Bacteria Kill *Caenorhabditis Elegans*: A New Model to Study Microbial-Microbial Interactions in the Gut. Am. J. Physiol Gastrointest. Liver Physiol..

[27] Beatty J.K., Akierman S.V., Motta J.-P., Muise S., Workentine M.L., Harrison J.J., Bhargava A., Beck P.L., Rioux K.P., McKnight G.W. (2017). Giardia Duodenalis Induces Pathogenic Dysbiosis of Human Intestinal Microbiota Biofilms. Int. J. Parasitol..

[28] Šlapeta J., Dowd S.E., Alanazi A.D., Westman M.E., Brown G.K. (2015). Differences in the Faecal Microbiome of Non-Diarrhoeic Clinically Healthy Dogs and Cats Associated with Giardia Duodenalis Infection: Impact of Hookworms and Coccidia. Int. J. Parasitol..

[29] Read C.M., Monis P.T., Andrew Thompson R.C. (2004). Discrimination of All Genotypes of Giardia Duodenalis at the Glutamate Dehydrogenase Locus Using PCR-RFLP. Infect. Genet. Evol..

[30] Sulaiman I.M., Fayer R., Bern C., Gilman R.H., Trout J.M., Schantz P.M., Das P., Lal A.A., Xiao L. (2003). Triosephosphate Isomerase Gene Characterization and Potential Zoonotic Transmission of *Giardia Duodenalis*. Emerg. Infect. Dis..

[31] Cacciò S.M., De Giacomo M., Pozio E. (2002). Sequence Analysis of the β-Giardin Gene and Development of a Polymerase Chain Reaction–Restriction Fragment Length Polymorphism Assay to Genotype Giardia Duodenalis Cysts from Human Faecal Samples. Int. J. Parasitol..

[32] Lalle M., Pozio E., Capelli G., Bruschi F., Crotti D., Cacciò S.M. (2005). Genetic Heterogeneity at the β-Giardin Locus among Human and Animal Isolates of Giardia Duodenalis and Identification of Potentially Zoonotic Subgenotypes. Int. J. Parasitol..

[33] Hopkins R.M., Meloni B.P., Groth D.M., Wetherall J.D., Reynoldson J.A., Thompson R.C. (1997). Ribosomal RNA Sequencing Reveals Differences between the Genotypes of Giardia Isolates Recovered from Humans and Dogs Living in the Same Locality. J. Parasitol..

[34] Nadkarni M.A., Martin F.E., Jacques N.A., Hunter N. (2002). Determination of Bacterial Load by Real-Time PCR Using a Broad-Range (Universal) Probe and Primers Set. Microbiology.

[35] Lluch J., Servant F., Païssé S., Valle C., Valière S., Kuchly C., Vilchez G., Donnadieu C., Courtney M., Burcelin R. (2015). The Characterization of Novel Tissue Microbiota Using an Optimized 16S Metagenomic Sequencing Pipeline. PLoS ONE.

[36] Heilmann R.M., Suchodolski J.S., Steiner J.M. (2008). Development and Analytic Validation of a Radioimmunoassay for the Quantification of Canine Calprotectin in Serum and Feces from Dogs. Am. J. Vet. Res..

[37] Godon J.J., Zumstein E., Dabert P., Habouzit F., Moletta R. (1997). Molecular Microbial Diversity of an Anaerobic Digestor as Determined by Small-Subunit RDNA Sequence Analysis. Appl. Environ. Microbiol..

[38] Escudié F., Auer L., Bernard M., Mariadassou M., Cauquil L., Vidal K., Maman S., Hernandez-Raquet G., Combes S., Pascal G. (2018). FROGS: Find, Rapidly, OTUs with Galaxy Solution. Bioinformatics.

[39] Cacciò S.M., Beck R., Lalle M., Marinculic A., Pozio E. (2008). Multilocus Genotyping of Giardia Duodenalis Reveals Striking Differences between Assemblages A and B. Int. J. Parasitol..

[40] Donowitz J.R., Alam M., Kabir M., Ma J.Z., Nazib F., Platts-Mills J.A., Bartelt L.A., Haque R., Petri W.A. (2016). A Prospective Longitudinal Cohort to Investigate the Effects of Early Life Giardiasis on Growth and All Cause Diarrhea. Clin. Infect. Dis..

[41] Hanevik K., Wensaas K.-A., Rortveit G., Eide G.E., Mørch K., Langeland N. (2014). Irritable Bowel Syndrome and Chronic Fatigue 6 Years After Giardia Infection: A Controlled Prospective Cohort Study. Clin. Infect. Dis..

[42] Riba A., Hassani K., Walker A., van Best N., von Zeschwitz D., Anslinger T., Sillner N., Rosenhain S., Eibach D., Maiga-Ascofaré O. (2020). Disturbed Gut Microbiota and Bile Homeostasis in *Giardia* -Infected Mice Contributes to Metabolic Dysregulation and Growth Impairment. Sci. Transl. Med..

[43] Kim M., Vogtmann E., Ahlquist D.A., Devens M.E., Kisiel J.B., Taylor W.R., White B.A., Hale V.L., Sung J., Chia N. (2020). Fecal Metabolomic Signatures in Colorectal Adenoma Patients Are Associated with Gut Microbiota and Early Events of Colorectal Cancer Pathogenesis. mBio.

[44] Perrucci S., Berrilli F., Procopio C., Di Filippo M.M., Pierini A., Marchetti V. (2020). Giardia Duodenalis infection in Dogs Affected by Primary Chronic Enteropathy. Open Vet. J..

[45] Hjøllo T., Bratland E., Steinsland H., Radunovic M., Langeland N., Hanevik K. (2018). Longitudinal Cohort Study of Serum Antibody Responses towards Giardia Lamblia Variant-Specific Surface Proteins in a Non-Endemic Area. Exp. Parasitol..

[46] Langford T.D., Housley M.P., Boes M., Chen J., Kagnoff M.F., Gillin F.D., Eckmann L. (2002). Central Importance of Immunoglobulin A in Host Defense against *Giardia* spp.. Infect. Immun..

[47] Bridgman S.L., Konya T., Azad M.B., Sears M.R., Becker A.B., Turvey S.E., Mandhane P.J., Subbarao P., Scott J.A., CHILD Study Investigators (2016). Infant Gut Immunity: A Preliminary Study of IgA Associations with Breastfeeding. J. Dev. Orig. Health Dis..

[48] Janzon A., Goodrich J.K., Koren O., Waters J.L., Ley R.E. (2019). Interactions between the Gut Microbiome and Mucosal Immunoglobulins A, M, and G in the Developing Infant Gut. mSystems.

[49] Paerewijck O., Maertens B., Gagnaire A., De Bosscher K., Geldhof P. (2019). Delayed Development of the Protective IL-17A Response Following a Giardia Muris Infection in Neonatal Mice. Sci. Rep..

[50] Ayling R.M., Kok K. (2018). Fecal Calprotectin. Advances in Clinical Chemistry.

[51] Ricciuto A., Griffiths A.M. (2019). Clinical Value of Fecal Calprotectin. Crit. Rev. Clin. Lab. Sci..

[52] Grellet A., Mila H., Heilmann R.M., Feugier A., Gruetzner N., Suchodolski J.S., Steiner J.M., Chastant-Maillard S. (2014). Effect of Age, Gestation and Lactation on Faecal IgA and Calprotectin Concentrations in Dogs. J. Nutr. Sci..

[53] Soto-Méndez M.-J., Romero-Abal M.-E., Schümann K., Gil Á., Solomons N.W. (2017). Normative Fecal Calprotectin Concentrations in Guatemalan Preschoolers Are High Relative to Children Reported Elsewhere. J. Pediatr. Gastroenterol. Nutr..

[54] Hestvik E., Tumwine J.K., Tylleskar T., Grahnquist L., Ndeezi G., Kaddu-Mulindwa D.H., Aksnes L., Olafsdottir E. (2011). Faecal Calprotectin Concentrations in Apparently Healthy Children Aged 0–12 Years in Urban Kampala, Uganda: A Community-Based Survey. BMC Pediatr..

[55] Hanevik K., Hausken T., Morken M.H., Strand E.A., Mørch K., Coll P., Helgeland L., Langeland N. (2007). Persisting Symptoms and Duodenal Inflammation Related to Giardia Duodenalis Infection. J. Infect..

[56] Odamaki T., Kato K., Sugahara H., Hashikura N., Takahashi S., Xiao J., Abe F., Osawa R. (2016). Age-Related Changes in Gut Microbiota Composition from Newborn to Centenarian: A Cross-Sectional Study. BMC Microbiol..

[57] Alessandri G., Argentini C., Milani C., Turroni F., Cristina Ossiprandi M., Sinderen D., Ventura M. (2020). Catching a Glimpse of the Bacterial Gut Community of Companion Animals: A Canine and Feline Perspective. Microb. Biotechnol..

[58] Omatsu T., Omura M., Katayama Y., Kimura T., Okumura M., Okumura A., Murata Y., Mizutani T. (2018). Molecular Diversity of the Faecal Microbiota of Toy Poodles in Japan. J. Vet. Med. Sci..

[59] Vilson Å., Ramadan Z., Li Q., Hedhammar Å., Reynolds A., Spears J., Labuda J., Pelker R., Björkstén B., Dicksved J. (2018). Disentangling Factors That Shape the Gut Microbiota in German Shepherd Dogs. PLoS ONE.

[60] Masuoka H., Shimada K., Kiyosue-Yasuda T., Kiyosue M., Oishi Y., Kimura S., Yamada A., Hirayama K. (2017). Transition of the Intestinal Microbiota of Dogs with Age. Biosci. Microbiota Food Health.

[61] Fallani M., Amarri S., Uusijarvi A., Adam R., Khanna S., Aguilera M., Gil A., Vieites J.M., Norin E., Young D. (2011). Determinants of the Human Infant Intestinal Microbiota after the Introduction of First Complementary Foods in Infant Samples from Five European Centres. Microbiology.

[62] Vallès Y., Artacho A., Pascual-García A., Ferrús M.L., Gosalbes M.J., Abellán J.J., Francino M.P. (2014). Microbial Succession in the Gut: Directional Trends of Taxonomic and Functional Change in a Birth Cohort of Spanish Infants. PLoS Genet..

[63] Martin-Gallausiaux C., Marinelli L., Blottière H.M., Larraufie P., Lapaque N. (2021). SCFA: Mechanisms and Functional Importance in the Gut. Proc. Nutr. Soc..

[64] Morrison D.J., Preston T. (2016). Formation of Short Chain Fatty Acids by the Gut Microbiota and Their Impact on Human Metabolism. Gut Microbes.

[65] Davis E.C., Dinsmoor A.M., Wang M., Donovan S.M. (2020). Microbiome Composition in Pediatric Populations from Birth to Adolescence: Impact of Diet and Prebiotic and Probiotic Interventions. Dig. Dis. Sci..

[66] Lee N.N., Bidot W.A., Ericsson A.C., Franklin C.L. (2020). Effects of Giardia Lamblia Colonization and Fenbendazole Treatment on Canine Fecal Microbiota. J. Am. Assoc. Lab. Anim. Sci..

[67] Mejia R., Damania A., Jeun R., Bryan P.E., Vargas P., Juarez M., Cajal P.S., Nasser J., Krolewiecki A., Lefoulon E. (2020). Impact of Intestinal Parasites on Microbiota and Cobalamin Gene Sequences: A Pilot Study. Parasit. Vectors.

[68] Yordanova I.A., Cortés A., Klotz C., Kühl A.A., Heimesaat M.M., Cantacessi C., Hartmann S., Rausch S. (2019). RORγt+ Treg to Th17 Ratios Correlate with Susceptibility to Giardia Infection. Sci. Rep..

[69] Atherly T., Rossi G., White R., Seo Y.-J., Wang C., Ackermann M., Breuer M., Allenspach K., Mochel J.P., Jergens A.E. (2019). Glucocorticoid and Dietary Effects on Mucosal Microbiota in Canine Inflammatory Bowel Disease. PLoS ONE.

[70] Pilla R., Suchodolski J.S. (2020). The Role of the Canine Gut Microbiome and Metabolome in Health and Gastrointestinal Disease. Front. Vet. Sci..

[71] Ley R.E. (2016). Prevotella in the Gut: Choose Carefully. Nat. Rev. Gastroenterol. Hepatol..

[72] Larsen J.M. (2017). The Immune Response to *Prevotella* Bacteria in Chronic Inflammatory Disease. Immunology.

[73] Iljazovic A., Roy U., Gálvez E.J.C., Lesker T.R., Zhao B., Gronow A., Amend L., Will S.E., Hofmann J.D., Pils M.C. (2021). Perturbation of the Gut Microbiome by *Prevotella* Spp. Enhances Host Susceptibility to Mucosal Inflammation. Mucosal Immunol..

[74] Rouhani S., Griffin N.W., Yori P.P., Olortegui M.P., Siguas Salas M., Rengifo Trigoso D., Moulton L.H., Houpt E.R., Barratt M.J., Kosek M.N. (2020). Gut Microbiota Features Associated With Campylobacter Burden and Postnatal Linear Growth Deficits in a Peruvian Birth Cohort. Clin. Infect. Dis..

[75] Toro-Londono M.A., Bedoya-Urrego K., Garcia-Montoya G.M., Galvan-Diaz A.L., Alzate J.F. (2019). Intestinal Parasitic Infection Alters Bacterial Gut Microbiota in Children. PeerJ.

[76] Hughes E.R., Winter M.G., Duerkop B.A., Spiga L., Furtado de Carvalho T., Zhu W., Gillis C.C., Büttner L., Smoot M.P., Behrendt C.L. (2017). Microbial Respiration and Formate Oxidation as Metabolic Signatures of Inflammation-Associated Dysbiosis. Cell Host Microbe.

[77] Litvak Y., Byndloss M.X., Tsolis R.M., Bäumler A.J. (2017). Dysbiotic Proteobacteria Expansion: A Microbial Signature of Epithelial Dysfunction. Curr. Opin. Microbiol..

[78] Lopez C.A., Miller B.M., Rivera-Chavez F., Velazquez E.M., Byndloss M.X., Chavez-Arroyo A., Lokken K.L., Tsolis R.M., Winter S.E., Baumler A.J. (2016). Virulence Factors Enhance Citrobacter Rodentium Expansion through Aerobic Respiration. Science.

[79] Rivera-Chávez F., Zhang L.F., Faber F., Lopez C.A., Byndloss M.X., Olsan E.E., Xu G., Velazquez E.M., Lebrilla C.B., Winter S.E. (2016). Depletion of Butyrate-Producing Clostridia from the Gut Microbiota Drives an Aerobic Luminal Expansion of Salmonella. Cell Host Microbe.

[80] Barash N.R., Maloney J.G., Singer S.M., Dawson S.C. (2017). Giardia Alters Commensal Microbial Diversity throughout the Murine Gut. Infect. Immun..

[81] Allain T., Fekete E., Buret A.G. (2019). Giardia Cysteine Proteases: The Teeth behind the Smile. Trends Parasitol..

[82] Bhargava A., Cotton J.A., Dixon B.R., Gedamu L., Yates R.M., Buret A.G. (2015). Giardia Duodenalis Surface Cysteine Proteases Induce Cleavage of the Intestinal Epithelial Cytoskeletal Protein Villin via Myosin Light Chain Kinase. PLoS ONE.

[83] Courtman N.F. (2016). Septic Peritonitis in a Dog Caused by *Anaerobiospirillum Succiniproducens*. Vet. Clin. Pathol..

[84] Epstein D.J., Ernst K., Rogers R., Carmody E., Aguero-Rosenfeld M. (2017). Closing the Brief Case: *Anaerobiospirillum Succiniciproducens* Bacteremia and Pyomyositis. J. Clin. Microbiol..

[85] Mehmood M., Jaffar N.A., Nazim M., Khasawneh F.A. (2014). Bacteremic Skin and Soft Tissue Infection Caused by *Prevotella Loescheii*. BMC Infect. Dis..

[86] Mondo E., Barone M., Soverini M., D’Amico F., Cocchi M., Petrulli C., Mattioli M., Marliani G., Candela M., Accorsi P.A. (2020). Gut Microbiome Structure and Adrenocortical Activity in Dogs with Aggressive and Phobic Behavioral Disorders. Heliyon.

[87] Yusof N., Hamid N., Ma Z.F., Lawenko R.M., Wan Mohammad W.M.Z., Collins D.A., Liong M.T., Odamaki T., Xiao J., Lee Y.Y. (2017). Exposure to Environmental Microbiota Explains Persistent Abdominal Pain and Irritable Bowel Syndrome after a Major Flood. Gut Pathog..

[88] Roskens H., Erlandsen S.L. (2002). Inhibition of In Vitro Attachment of Giardia Trophozoites by Mucin. J. Parasitol..

[89] Yan S., Yang B., Zhao J., Zhao J., Stanton C., Ross R.P., Zhang H., Chen W. (2019). A Ropy Exopolysaccharide Producing Strain *Bifidobacterium Longum* Subsp. *Longum* YS108R Alleviates DSS-Induced Colitis by Maintenance of the Mucosal Barrier and Gut Microbiota Modulation. Food Funct..

[90] Praharaj A.B., Dehury B., Mahapatra N., Kar S.K., Behera S.K. (2018). Molecular Dynamics Insights into the Structure, Function, and Substrate Binding Mechanism of Mucin Desulfating Sulfatase of Gut Microbe Bacteroides Fragilis. J. Cell. Biochem..

[91] Rho J., Wright D.P., Christie D.L., Clinch K., Furneaux R.H., Roberton A.M. (2005). A Novel Mechanism for Desulfation of Mucin: Identification and Cloning of a Mucin-Desulfating Glycosidase (Sulfoglycosidase) from Prevotella Strain RS2. J. Bacteriol..

[92] Amat C.B., Motta J.-P., Fekete E., Moreau F., Chadee K., Buret A.G. (2017). Cysteine Protease–Dependent Mucous Disruptions and Differential Mucin Gene Expression in *Giardia duodenalis* Infection. Am. J. Pathol..

[93] Halliez M.C.M., Motta J.-P., Feener T.D., Guérin G., LeGoff L., François A., Colasse E., Favennec L., Gargala G., Lapointe T.K. (2016). *Giardia Duodenalis* Induces Paracellular Bacterial Translocation and Causes Postinfectious Visceral Hypersensitivity. Am. J. Physiol. Gastrointest. Liver Physiol..

[94] Allain T., Amat C.B., Motta J.-P., Manko A., Buret A.G. (2017). Interactions of *Giardia Sp.* with the Intestinal Barrier: Epithelium, Mucus, and Microbiota. Tissue Barriers.

[95] Manko-Prykhoda A., Allain T., Motta J.-P., Cotton J.A., Feener T., Oyeyemi A., Bindra S., Vallance B.A., Wallace J.L., Beck P. (2020). *Giardia* spp. Promote the Production of Antimicrobial Peptides and Attenuate Disease Severity Induced by Attaching and Effacing Enteropathogens via the Induction of the NLRP3 Inflammasome. Int. J. Parasitol..

[96] Martínez C., Lasitschka F., Thöni C., Wohlfarth C., Braun A., Granzow M., Röth R., Dizdar V., Rappold G.A., Hausken T. (2020). Comparative Expression Profiling in the Intestine of Patients with *Giardia* -induced Postinfectious Functional Gastrointestinal Disorders. Neurogastroenterol. Motil..

[97] Cerquetella M., Rossi G., Spaterna A., Tesei B., Jergens A., Suchodolski J., Bassotti G. (2018). Is irritable bowel syndrome also present in dogs?. Tierärztl. Prax. Ausg. K Kleintiere Heimtiere.

[98] Pei L., Ke Y., Zhao H., Wang L., Jia C., Liu W., Fu Q., Shi M., Cui J., Li S. (2019). Role of Colonic Microbiota in the Pathogenesis of Ulcerative Colitis. BMC Gastroenterol..

[99] Jalanka J., Cheng J., Hiippala K., Ritari J., Salojärvi J., Ruuska T., Kalliomäki M., Satokari R. (2020). Colonic Mucosal Microbiota and Association of Bacterial Taxa with the Expression of Host Antimicrobial Peptides in Pediatric Ulcerative Colitis. Int. J. Mol. Sci..

[100] Lavelle A., Lennon G., O’Sullivan O., Docherty N., Balfe A., Maguire A., Mulcahy H.E., Doherty G., O’Donoghue D., Hyland J. (2015). Spatial Variation of the Colonic Microbiota in Patients with Ulcerative Colitis and Control Volunteers. Gut.

[101] Chung C.-S., Chang P.-F., Liao C.-H., Lee T.-H., Chen Y., Lee Y.-C., Wu M.-S., Wang H.-P., Ni Y.-H. (2016). Differences of Microbiota in Small Bowel and Faeces between Irritable Bowel Syndrome Patients and Healthy Subjects. Scand. J. Gastroenterol..

[102] Masoodi I., Alshanqeeti A.S., Alyamani E.J., AlLehibi A.A., Alqutub A.N., Alsayari K.N., Alomair A.O. (2020). Microbial Dysbiosis in Irritable Bowel Syndrome: A Single-center Metagenomic Study in Saudi Arabia. JGH Open.

[103] Tana C., Umesaki Y., Imaoka A., Handa T., Kanazawa M., Fukudo S. (2009). Altered Profiles of Intestinal Microbiota and Organic Acids May Be the Origin of Symptoms in Irritable Bowel Syndrome. Neurogastroenterol. Motil..

[104] Lo Presti A., Zorzi F., Del Chierico F., Altomare A., Cocca S., Avola A., De Biasio F., Russo A., Cella E., Reddel S. (2019). Fecal and Mucosal Microbiota Profiling in Irritable Bowel Syndrome and Inflammatory Bowel Disease. Front. Microbiol..

[105] Enqi W., Jingzhu S., Lingpeng P., Yaqin L. (2021). Comparison of the Gut Microbiota Disturbance in Rat Models of Irritable Bowel Syndrome Induced by Maternal Separation and Multiple Early-Life Adversity. Front. Cell. Infect. Microbiol..

[106] Haange S.-B., Jehmlich N., Hoffmann M., Weber K., Lehmann J., von Bergen M., Slanina U. (2019). Disease Development Is Accompanied by Changes in Bacterial Protein Abundance and Functions in a Refined Model of Dextran Sulfate Sodium (DSS)-Induced Colitis. J. Proteome Res..

[107] Wang C.-S.-E., Li W.-B., Wang H.-Y., Ma Y.-M., Zhao X.-H., Yang H., Qian J.-M., Li J.-N. (2018). VSL#3 Can Prevent Ulcerative Colitis-Associated Carcinogenesis in Mice. World J. Gastroenterol..

[108] Suchodolski J.S., Markel M.E., Garcia-Mazcorro J.F., Unterer S., Heilmann R.M., Dowd S.E., Kachroo P., Ivanov I., Minamoto Y., Dillman E.M. (2012). The Fecal Microbiome in Dogs with Acute Diarrhea and Idiopathic Inflammatory Bowel Disease. PLoS ONE.

[109] Chen Y.-J., Wu H., Wu S.-D., Lu N., Wang Y.-T., Liu H.-N., Dong L., Liu T.-T., Shen X.-Z. (2018). *Parasutterella*, in Association with Irritable Bowel Syndrome and Intestinal Chronic Inflammation: *Parasutterella* May Be Related with IBS. J. Gastroenterol. Hepatol..

[110] Chiodini R.J., Dowd S.E., Chamberlin W.M., Galandiuk S., Davis B., Glassing A. (2015). Microbial Population Differentials between Mucosal and Submucosal Intestinal Tissues in Advanced Crohn’s Disease of the Ileum. PLoS ONE.

[111] Bloom S.M., Bijanki V.N., Nava G.M., Sun L., Malvin N.P., Donermeyer D.L., Dunne W.M., Allen P.M., Stappenbeck T.S. (2011). Commensal Bacteroides Species Induce Colitis in Host-Genotype-Specific Fashion in a Mouse Model of Inflammatory Bowel Disease. Cell Host Microbe.

[112] Chao G., Zhang S. (2020). The Characteristics of Intestinal Flora of IBS-D with Different Syndromes. Immun. Inflamm. Dis..

[113] Pittayanon R., Lau J.T., Yuan Y., Leontiadis G.I., Tse F., Surette M., Moayyedi P. (2019). Gut Microbiota in Patients With Irritable Bowel Syndrome—A Systematic Review. Gastroenterology.

[114] Allain T., Chaouch S., Thomas M., Vallée I., Buret A.G., Langella P., Grellier P., Polack B., Bermúdez-Humarán L.G., Florent I. (2018). Bile-Salt-Hydrolases from the Probiotic Strain Lactobacillus Johnsonii La1 Mediate Anti-Giardial Activity in Vitro and in Vivo. Front. Microbiol..

[115] Alazzaz J., Chaouch S., Boucard A.-S., Bermudez-Humaran L.G., Florent I., Guillen N. (2020). Probiotics as Anti-Giardia Defenders: Overview on Putative Control Mechanisms. Eukaryome Impact on Human Intestine Homeostasis and Mucosal Immunology.

[116] Kodio A., Coulibaly D., Koné A.K., Konaté S., Doumbo S., Guindo A., Bittar F., Gouriet F., Raoult D., Thera M.A. (2019). Blastocystis Colonization Is Associated with Increased Diversity and Altered Gut Bacterial Communities in Healthy Malian Children. Microorganisms.

[117] Audebert C., Even G., Cian A., Loywick A., Merlin S., Viscogliosi E., Chabé M., The Blastocystis Investigation Group (2016). Colonization with the Enteric Protozoa Blastocystis Is Associated with Increased Diversity of Human Gut Bacterial Microbiota. Sci. Rep..

[118] Allain T., Buret A.G. (2020). Pathogenesis and post-infectious complications in giardiasis. Advances in Parasitology.

