# Supplementary material for: Age and Giardia intestinalis Infection Impact Canine Gut Microbiota
Source: Microorganisms. 2021 Sep 2;9(9):1862. doi: 10.3390/microorganisms9091862 (PMC8469385; doi:10.3390/microorganisms9091862)
Supplement: Supplementary file 1 [file microorganisms-09-01862-s001.zip › microorganisms-1330156-supplementary.pdf]

**Supplementary Materials:**

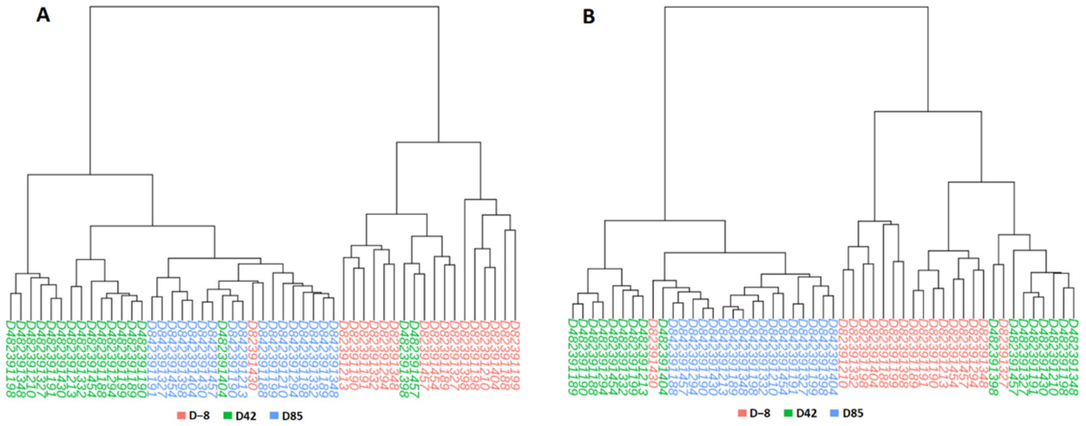


**Figure S1**. Beta diversity. Sample clustering using (**A**) Jaccard or (**B**) unweight Unifrac beta diversity
